# Songs and morphology in three species of the *Chorthippusbiguttulus* group (Orthoptera, Acrididae, Gomphocerinae) in Russia and adjacent countries

**DOI:** 10.3897/zookeys.1073.75539

**Published:** 2021-11-29

**Authors:** Tatiana Tarasova, Dmitry Tishechkin, Varvara Vedenina

**Affiliations:** 1 Institute for Information Transmission Problems, Russian Academy of Sciences, Bolshoy Karetny per.19, Moscow 127051 Russia Institute for Information Transmission Problems, Russian Academy of Sciences Moscow Russia; 2 Department of Entomology, Faculty of Biology, Moscow State University, Leninskie Gory, Moscow 119234 Russia Moscow State University Moscow Russia

**Keywords:** Calling song, courtship song, grasshoppers, leg-movement pattern, rivalry song, stridulatory file

## Abstract

Songs and morphology are compared between *Chorthippusmiramae* (Vorontsovsky, 1928) that was previously named as *C.porphyropterus* and two other closely related species, *C.brunneus* (Thunberg, 1815) and *C.maritimus* Mistshenko, 1951. We compare them because the calling song of *C.miramae* was previously shown to have song elements similar to those of other two species. One morphological character, the length of stridulatory file, appeared to be the best character to distinguish between all three species. For *C.maritimus* and *C.miramae*, we present the morphological descriptions since they are absent in the literature. We also establish the synonymy *C.maritimus = C.bornhalmi* Harz, 1971, **syn. n.** = *C.biguttuluseximius* Mistshenko, 1951, **syn. n.** In the song analysis, we analyse not only the sound but also the leg-movement pattern, which is very helpful to find a homology between various song elements. We show that the calling song of *C.miramae* usually contains two elements, one element being similar to the *C.brunneus* calling song, and another – to the *C.maritimus* calling song. Despite some similarities, the calling song elements in *C.miramae* have some peculiarities. The courtship song of *C.miramae* is similar to the *C.brunneus* song, whereas the rivalry songs of *C.miramae* comprise both the *maritimus*-like elements and the unique ones. *C.miramae* generally demonstrates a richer song repertoire than the other two species.

## Introduction

In singing Orthoptera, the song is an important component of reproductive isolation. Acoustic signals are often used in taxonomy, when sibling species are similar in morphology, but different in songs. In grasshoppers of subfamily Gomphocerinae, the song is produced by stroking the stridulatory file of each hind femur across a raised vein on the fore wing. It is noteworthy that using both hind legs, the grasshoppers have two separate sound-producing devices, which must be coordinated with one another. The stridulatory movements of the two legs often differ in amplitude and pattern, and the legs can exchange roles from time to time, which leads to an increase of song complexity (e.g., Elsner 1974; Helversen and Elsner 1977; Helversen and Helversen 1994). To distinguish cryptic grasshopper species, not only the sound recordings but also the recordings of the leg movements are used by various authors (Helversen 1986; Gottsberger and Mayer 2007; Vedenina and Helversen 2003, 2009; Willemse et al. 2009; Vedenina et al. 2012; Tarasova et al. 2021).

Closely related grasshopper species belonging to the *Chorthippusbiguttulus* group offer an excellent example of the cryptic species complex that can only be reliably identified by the male calling songs (Ragge and Reynolds 1988, 1998; Helversen 1989; Ragge et al. 1990; Bukhvalova 1993, 1998; Ingrisch 1995; Willemse et al. 2009; Sirin et al. 2010). This group includes four species with large ranges across Europe and Asia: *C.biguttulus* (Linnaeus, 1758), *C.brunneus* (Thunberg, 1815), *C.mollis* (Charpentier, 1825), and *C.maritimus* Mistshenko, 1951. Other species of this group with smaller ranges occur in southern Europe, namely, *C.jacobsi* Harz, 1975 and *C.yersini* Harz, 1975 in the Iberian Peninsula, *C.rubratibialis* Schmidt, 1978 in Italy and *C.bornhalmi* Harz, 1971 in the Balkans. Two additional species are endemic to Greece (Willemse et al. 2009) and two more to Anatolia (Sirin et al. 2010). Several species and subspecies only occur in Russia and adjacent territories, in particular, *C.porphyropterus* (Vorontsovsky 1928) (Benediktov 1999, 2005).

The main subject of the current study is one species of the *biguttulus* group, *C.porphyropterus*, which we name as *C.miramae* (Vorontsovsky, 1928 nec Ramme, 1936, 1951), and two closely related species, *C.brunneus* and *C.maritimus*, whose songs resemble song elements of *C.miramae*. Since in Russia and adjacent countries *C.brunneus*, *C.maritimus* and *C.miramae* often occur with two other species of the *biguttulus* group, *C.biguttulus* and *C.mollis*, we describe the main morphological differences from the latter two species as well.

## Materials and methods

**Figure 1.**
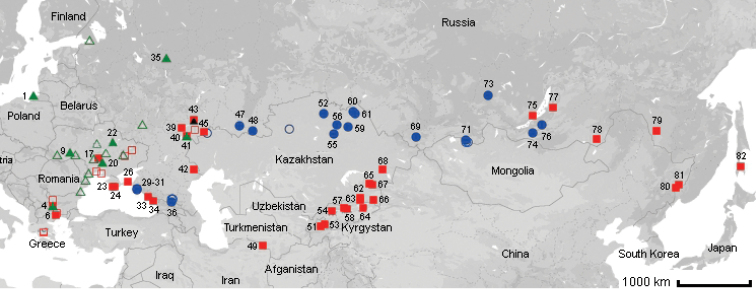
Map of localities where the specimens of *Chorthippusbrunneus* (green triangles), *C.maritimus* (red squares) and *C.miramae* (blue circles) were collected. The localities with song recordings are numbered and marked by filled icons.

Localities where material was collected are shown in Fig. 1. All localities were numbered and all numbers are listed in Results, in the paragraph “Material examined”. On the map, however, only localities with song recordings are numbered.

### Morphological analysis

**Figure 2.**
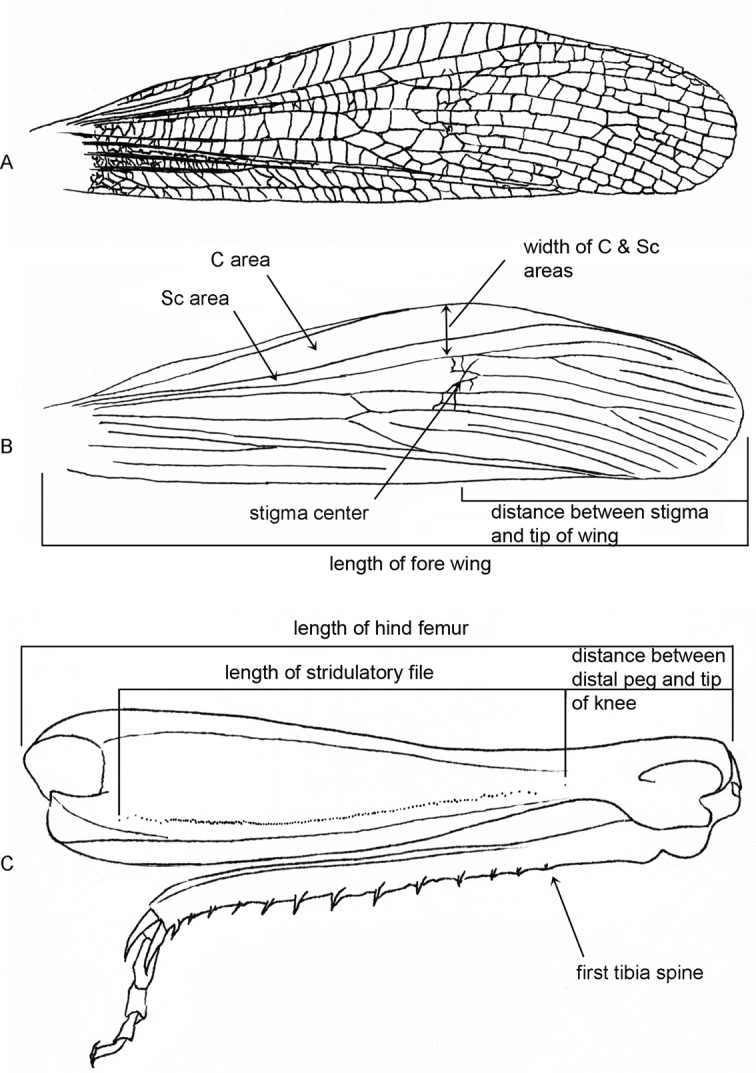
Morphology of fore wing and hind leg in *Chorthippusmiramae* (Vorontsovsky) from Orenburg region **A** fore wing with complete venation **B** fore wing with main veins; **C** hind leg. The measured morphological characters are indicated with arrows and brackets.

In all specimens studied, we measured the following morphological characters: the lengths of pronotum, forewing and hind femur, the width of costal and subcostal (C & Sc) areas of fore wing, the distance from the center of stigma to the tip of fore wing, the length of stridulatory file and the distance from the most distal stridulatory peg to the tip of knee (Table 1, Fig. 2). In 10 specimens of each sex and species, the body length, the width of fore wing and the number of stridulatory pegs were measured. These morphological features have been chosen on the basis of the literature (Ragge et al. 1988; Bukhvalova 1993; Benediktov 1999; Willemse et al. 2009).The length of pronotum was measured along the midline. The length of forewing was measured from the humeral plate to the tip of the wing; the widths of the C & Sc areas were measured at the point where costal area was of the greatest width (Fig. 2B). The length of hind femur was measured from the anterior margin of the upper basal lobe to the hind margin of the upper knee-lobe; the length of stridulatory file was measured from the most proximal peg to the most distal peg; the distance between stridulatory file to the tip of the knee was measured from the most distal peg to the hind margin of the upper knee-lobe (Fig. 2C). Morphological studies were carried out with an MBS-9 light microscope at 8–56× magnification using an ocular micrometer. Material for the morphological analysis was taken from the Zoological Museum of Moscow State University (ZMMU) and the personal collections of V. Vedenina (CV).

**Table 1.** Morphological measurements in three species of the *Chorthippusbiguttulus* group. For each character, mean, standard deviation, min and max are shown. Abbreviations in brackets see in Fig. 2.

Number of specimens	Males			Females		
	*C.miramae*	*C.maritimus*	*C.brunneus*	*C.miramae*	*C.maritimus*	*C.brunneus*
	133	122	53	50	28	35
Length of pronotum, mm	3.14±0.25 2.60–3.70	3.25±0.23 2.80–3.60	3.06±0.15 2.80–3.50	4.17±0.36 3.50–4.90	4.26 ±0.25 3.80–4.80	3.89±0.27 3.40–4.40
Length of fore wing, mm	14.06±0.89 12.10–16.30	14.87±1.09 12.50–16.60	14.30±0.82 12.40–15.70	17.49±1.46 12.40–19.90	17.78±1.18 15.20–20.70	17.12±1.29 14.30–19.20
Length from stigma to tip of fore wing, mm	6.07±0.64 4.30–7.70	5.90±0.69 4.50–7.30	5.61±0.38 4.60–6.20	8.38±0.73 6.60–10.80	7.47±0.97 5.80–9.90	6.75±0.99 4.10–8.30
Width of C & Sc areas, mm	10.53±0.96 7.00–13.00	9.52±0.84 7.50–11.00	9.05±0.61 7.50–10.00	7.58±0.65 6.00–9.00	7.20±0.75 6.00–9.00	7.10±0.64 6.00–9.00
Length of hind femur, mm	10.09±0.58 8.90–11.7	10.21±0.61 9.20–12.80	9.57±0.41 8.70–10.70	13.21±1.09 9.30–15.20	13.48±0.99 11.90–15.40	12.21±0.99 10.20–14.40
Length of stridulatory file, mm	5.78±0.87 3.10–7.45	4.41±0.55 3.40–6.30	3.13±0.25 2.70–3.90	7.35±0.76 5.70–9.20	5.45±1.04 3.50–8.50	4.29±0.99 3.10–7.70
Length from last distal peg to tip of knee, mm	2.73±0.69 1.60–5.05	4.11±0.55 2.20–5.50	4.74±0.29 4.20–5.40	3.63±0.57 2.60–4.90	5.63±0.69 4.10–7.00	5.75±1.00 2.30–7.40

**Figure 3.**
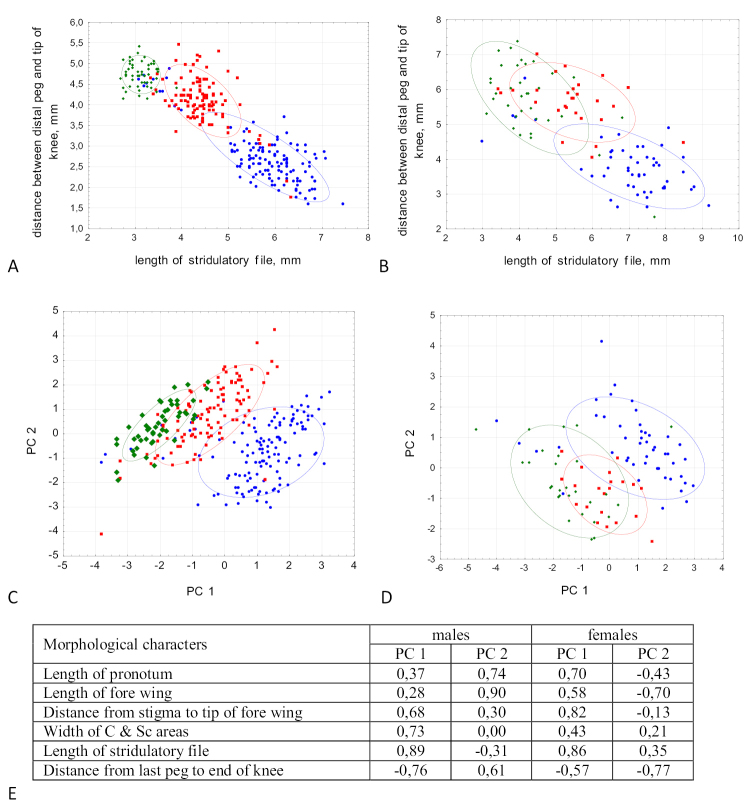
Morphological differences between *Chorthippusbrunneus* (green dots), *C.maritimus* (red dots), and *C.miramae* (blue dots). **A,B** length of stridulatory file vs. distance from the last stridulatory peg to the tip of knee in males (**A**) and females (**B**) **C,D** results of Principal Component Analysis based on 6 characters are shown for PC 1 and PC 2 in males (**C**) and females (**D**) **E** loadings of different characters to PC 1 and PC 2.

All statistical analyses were performed using Excel 2016 and STATISTICA 12.0.0. To visualize and clarify the differences in morphology between the three species, a principal component analysis (PCA) was applied to 6 morphological characters (Fig. 3E).

### Song recordings and analysis

The calling song was recorded from an isolated male; the courtship song was recorded when a male was sitting near a female; the rivalry song was recorded from males sitting near each other. Recordings of the calling and rivalry songs in the field were carried out with a MD-382 microphone (upper frequency limit 12.5 kHz; before 2008), or a Spirit IM-01 microphone (upper frequency limit 20 kHz), and an Elektronika-302-1 cassette recorder (upper frequency limit 10 kHz; before 2005), or a Sony Walkman MZ-NH900 minidisk recorder (sampling frequency 44.1 kHz). The signals were A/D converted with a PC card L-305 (L-Card Ltd., Russia). The ambient temperature near a singing male in the field was 20–40°C.

During stridulation of the males studied in laboratory, both the sound and the hind leg movements were recorded with a custom-built opto-electronic device (Helversen and Elsner 1977; Hedwig 2000). A piece of reflecting foil was glued to the distal part of each hind leg femur of a male and two opto-electronic cameras were focused on the illuminated reflecting dots. Each camera was equipped with a position-sensitive photodiode that converted the upward and downward movements of the hind legs into voltage signals. These signals, together with the recordings of the sounds (a microphone type 4191, ½ inch; a conditioning amplifier type 2690; Brüel & Kjaer, Nærum, Denmark), were A/D-converted with a custom-built PC card. The sampling rate was 1325 Hz for recording the stridulatory movements and 100 kHz for sound recordings. In the laboratory, the ambient temperature near a singing male was 30–32°C.

All recordings were analyzed with COOLEDIT 2.0 (Syntrillium, Seattle, WA) and TURBOLAB 4.0 (Bressner Technology, Gröbenzell, Germany). All statistical analyses were performed using Excel 2016 and STATISTICA 12.0.0.

**Figure 4.**
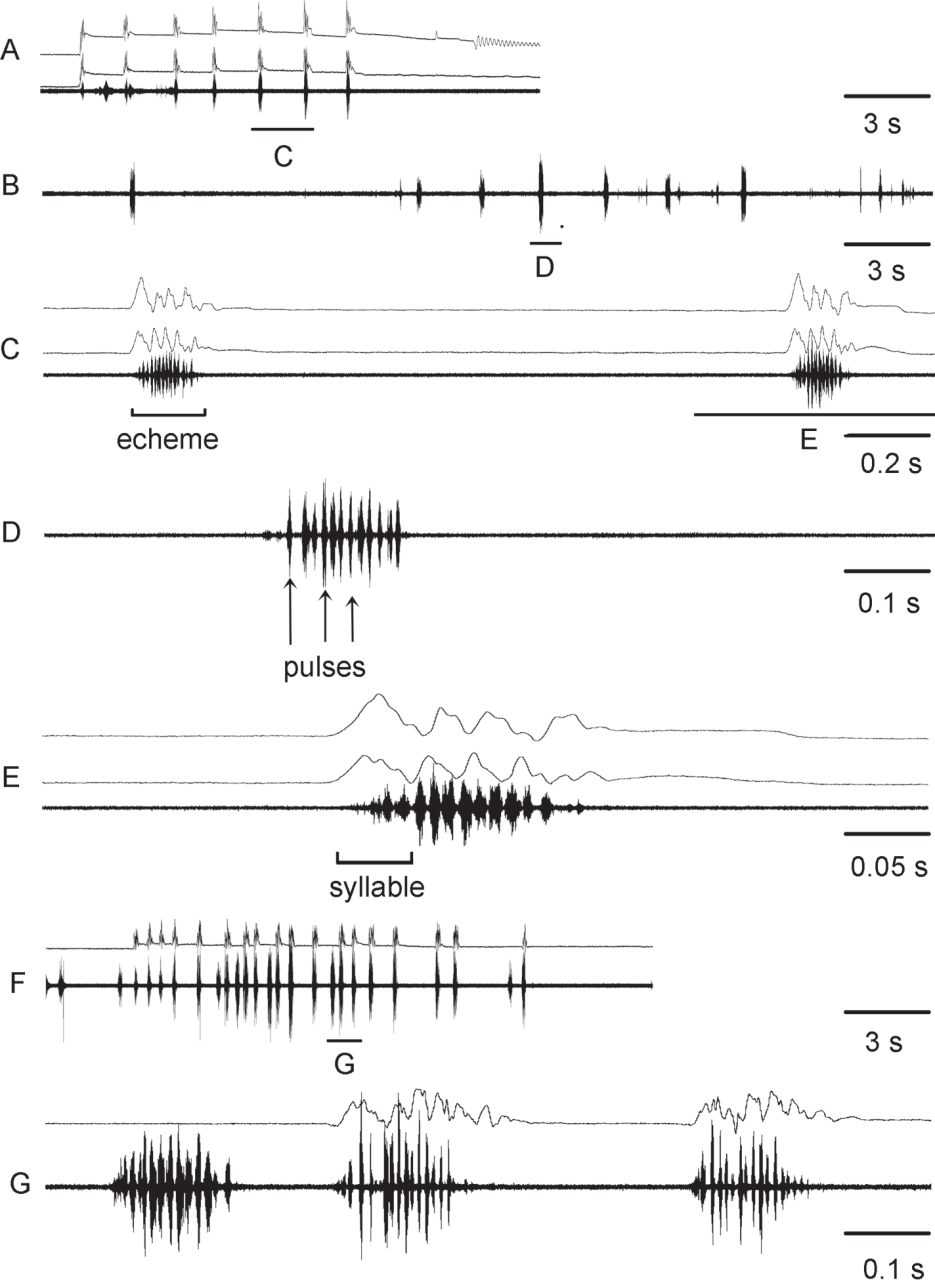
Oscillograms of calling songs **A–E** and rivalry songs **F,G** in *Chorthippusbrunneus* from Kostroma region (**A**) Poltava region (**B**) and Saratov region (**F**). Song recordings are presented at four different speeds (faster oscillograms of the indicated parts of the songs shown in **C,D,E,G**). In all oscillograms the two upper lines are recordings of hind leg movements and the lower line is the sound recording. Different song parameters are indicated by brackets and arrows. The ambient temperature near a singing male was 29 – 32°C.

**Figure 5.**
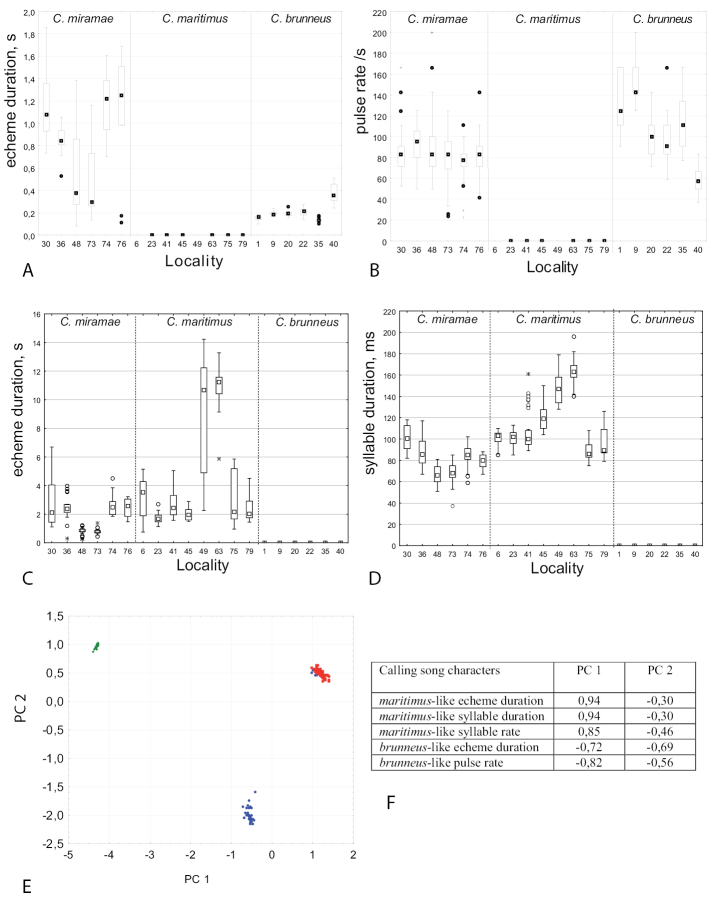
Differences in calling songs between *Chorthippusbrunneus*, *C.maritimus*, and *C.miramae***A–D** boxplots for the *brunneus*-like echeme duration (**A**) for the *brunneus*-like pulse rate (**B**) for the *maritimus*-like echeme duration (**C**) and the *maritimus*-like syllable duration (**D**) medians (dots), first and third quartiles (boxes), the 10^th^ and 90^th^ percentiles (whiskers), and outliers (dots beyond whiskers) are shown **E** results of Principal Component Analysis based on 5 song characters are shown for PC 1 and PC 2 in *C.brunneus* (green dots), *C.maritimus* (red dots), and *C.miramae* (blue dots) **F** loadings of different characters to PC 1 and PC 2.

**Figure 6.**
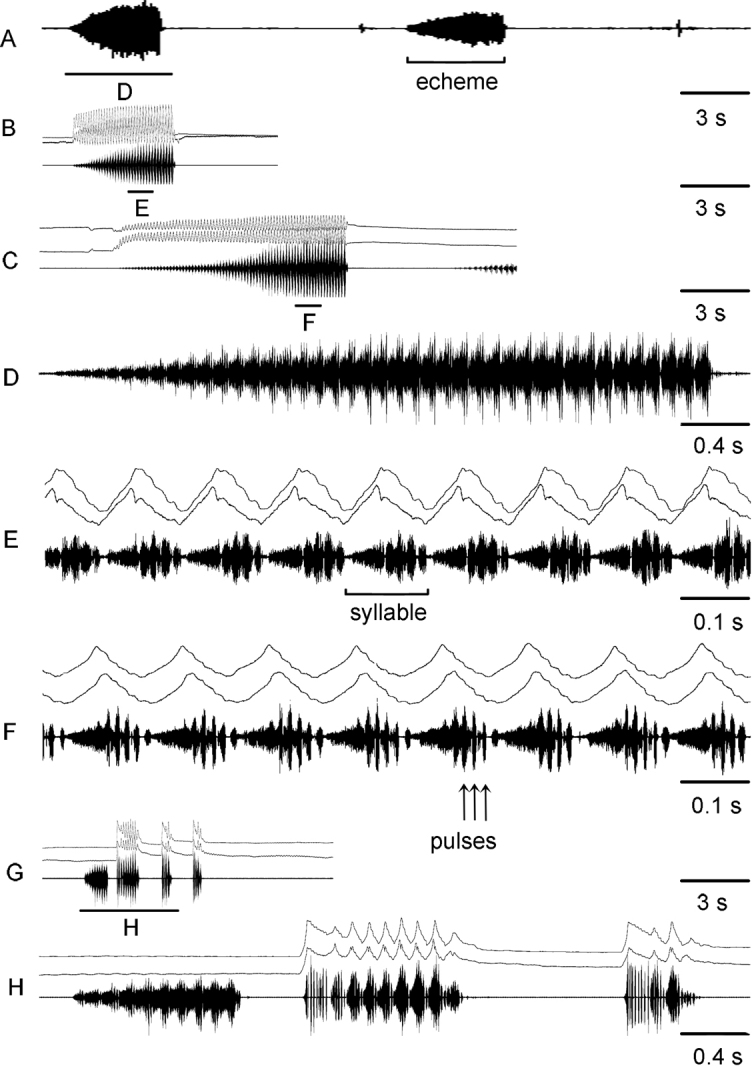
Oscillograms of calling songs **A–F** and rivalry songs **G,H** in *Chorthippusmaritimus* from Primorskiy kray (**A**) Macedonia (**B**) Sukhumi region (**C**) and Saratov region (**G**). Song recordings are presented at three different speeds (faster oscillograms of the indicated parts of the songs shown in **D,E,F,H**). In all oscillograms the two upper lines are recordings of hind leg movements and the lower line is the sound recording. The ambient temperature near a singing male was 33 – 34°C in (**A**) and 29 – 30°C in other cases.

For the song description we used the following terminology (Figs 4, 6): *pulse* – the sound produced by one stroke of a hind leg (the shortest measurable unit or the first-order unit); *syllable* – the sound produced by one complete up and down movement of the hind legs, starting when the legs leave their initial position and ending when the legs return to their original position and representing the repeated unit of a stable structure (the second-order unit); *echeme* – series of consistent syllables separated by pauses (the third-order unit). We measured three characters in *C.brunneus* (echeme rate, echeme duration and pulse rate), four characters in *C.maritimus* (echeme rate and duration and syllable rate and duration) and seven characters in *C.miramae* (echeme rate, echeme duration and pulse rate for the *brunneus*-like echeme and echeme rate and duration and syllable rate and duration for the *maritimus*-like echeme). To visualize and clarify the differences in calling song between the three species, a PCA was applied to 5 song characters (Fig. 5E). We did not use echeme rate for both types of echemes because not all recorded males produced several echemes. When a character was equal to 0, we changed it to 0.01 by convention because we only used the logarithmic values for PCA.

## Results

### Nomenclatural notes

The names *Stauroderusmollisporphyroptera* and *S.miramae* (both currently included in the subgenus Glyptobothrus Chopard, 1951) were described by Vorontsovsky (1928a, b) in two papers on grasshoppers from Orenburg published in the same issue. *S.mollisporphyroptera* was described as a new variation and designated as a var. nov.; therefore, the authorship of Vorontsovsky in this case is beyond doubt (Vorontsovsky 1928a, p. 12). Vorontsovsky attributed the authorship of the *S.miramae* to Ramme, with the following comment: “For the identification of this species, as well as the form, I identified as a variety of the species *Stauroderusmollis*, I take the opportunity to express here my deep gratitude to E.F. Miram, who informed me that *S.miramae* has just been described from Crimea by Dr. Ramme as a new species.” (Vorontsovsky 1928a: 12, footnote). Actually, Ramme mentioned *Chorthippusmiramae* for the first time only in 1939 without a description, specifying that this species “will be described in the near future” (Ramme 1939: 131). Therefore, the name *C.miramae* Ramme, 1939 is suggested to be a *nomen nudum*. Only in 1951, Ramme described this species based on material from Ukraine, Crimea, Southern and South-eastern European Russia, Caucasus, and Transcaucasia, with the type locality in Southern Crimea (Ramme 1951). On the other hand, Vorontsovsky (1928b) presented a short description of *C.miramae*. For this reason, he is the author of the taxon from Orenburg in spite of the fact that he attributed the authorship to Ramme. Further, the study of signals showed that *C.miramae* Vorontsovsky and *C.miramae* Ramme represent the different species (see below).

Summarizing the following three taxa were described in the papers mentioned above: *C.mollisporphyroptera* (Vorontsovsky 1928) from the type locality in Orenburg, *C.miramae* (Vorontsovsky, 1928) from the type locality in Orenburg, and *C.miramae* Ramme, 1951 from the type locality in Southern Crimea.

According to the study of Bukhvalova (1993) based on investigation of the male songs, the *Chorthippusbiguttulus* group includes 5 species in Russia: *C.biguttulus* (Linnaeus, 1758), *C.brunneus* (Thunberg, 1815), *C.mollis* (Charpentier, 1825), *C.miramae* Ramme, 1939 and *C.yersini* Harz, 1975. The study of the songs of specimens from Crimea, Southern European Russia, North Caucasus, Central Asia, and the Russian Far East showed that *C.miramae* Ramme, 1939 *sensu*Bukhvalova (1993) is a good species, which is widespread throughout the southern part of Russia and adjacent territories. It was described as *C.biguttulusmeridionalis* Mistshenko, 1950 from mountains of Central Asia (Mistshenko 1950), as *C.miramae* Ramme, 1951 from Crimea, and as *C.maritimus* Mistshenko, 1951 from the Russian Far East (Bey-Bienko and Mistshenko 1951). However, it differs from the taxa described by Vorontsovsky (1928a, b) from Orenburg (Bukhvalova 1998). The name *C.biguttulusmeridionalis* Mistshenko, 1950 is invalid, since it is a junior homonym of C.bicolorvar.meridionalis (Fruhstorfer, 1921). The name *C.miramae* Ramme, 1951 is a junior homonym of *C.miramae* (Vorontsovsky, 1928). As a result, the valid name of this taxon should be *C.maritimus* Mistshenko, 1951. It should be also noted that some authors improperly considered the date of publication of the name *C.miramae* Ramme to be 1939 (Bey-Bienko and Mistshenko 1951; Harz 1975; Bukhvalova 1993; Wosnessenskij 1996) and treated this taxon as a subspecies of *C.brunneus* (Bey-Bienko and Mistshenko 1951; Harz 1975).

*C.bornhalmi* Harz, 1971 was described from Croatia in the Balkans and has been shown to occur from Italy to Turkey (Willemse et al. 2009; Sirin et al. 2010). The range of *C.maritimus* extends from southern Ukraine to the Russian Far East. In the current study, we compare the morphology and songs in *C.bornhalmi* (from Bulgaria and Greece) and *C.maritimus*, and establish the synonymy *C.maritimus* Mistshenko, 1951 = *C.bornhalmi* Harz, 1971, syn. n.

*C.biguttuluseximius* Mistshenko, 1951 was described from Sukhumi, Abkhazia (Mistshenko 1950). A study of songs from the environs of the type locality (loc. 34 in Fig. 1) showed that this subspecies also is identical to *C.maritimus*. Since *C.maritimus* (as *C.biguttulusmaritimus*) and *C.biguttuluseximius* were described in the same paper, we choose a valid name *C.maritimus* for this species and establish the synonymy *C.maritimus = C.biguttuluseximius* syn.n.

*C.miramaellus* Wosnessenskij, 1996 and *C.sinuatus* Mistshenko and Wosnessenskij, 1996 proposed by Wosnessenskij (1996) to replace *C.miramae* Ramme, 1951 and *C.biguttulusmeridionalis* Mistshenko, 1950 respectively, are the junior synonyms of *C.maritimus* (Bukhvalova 1998). We suggest that *C.maritimustsejensis* Bukhvalova, 1993 from North Ossetia, North Caucasus (Bukhvalova 1993) and *C.meridionaliskarakalensis* Sytshev and Woznesenskij, 1995 from South-western Turkmenistan (Sychov and Voznesensky 1995) also belong to *C.maritimus*; however, additional studies are needed to clarify their status. It should be noted that M.M. Sychov and A.Yu. Voznesensky transliterated their own names in different ways in different papers, both in English and Latin; here we present their original spellings from the corresponding papers.

Benediktov (1999) reinvestigated material from Orenburg used by Vorontsovsky and concluded that *C.mollisporphyroptera* (Vorontsovsky, 1928) and *C.miramae* (Vorontsovsky, 1928) are synonyms. Benediktov (1999) compared the lengths of stridulatory files (the most characteristic feature of this species) in the type specimens of Vorontsovsky and found them to be identical. He proposed *C.porphyropterus* as the valid name, raising its rank, and changing its gender ending. However, according to chapter 24 of the International Code of Zoological Nomenclature (1999), when synonyms are established simultaneously, but are proposed at different ranks, the name proposed at a higher rank takes precedence. Consequently, the valid name of the taxon from Orenburg should be *C.miramae* (Vorontsovsky, 1928). Also, Benediktov (1999) established the synonymy *C.porphyropterus = C.biguttulus* forma tomensis Berezhkov, 1956, proposed the new combination *C.porphyropteruseuchedickei* Helversen, 1989 for *C.biguttuluseuchedickei* Helversen, 1989, and pointed out that *C.yersini* Harz, 1975 sensu Bukhvalova, 1993 is conspecific with *C.miramae* (Vorontsovsky, 1928). The true identity of C.biguttulusformatomensis described known only from the bank of the Tom’ River near Ust’-Iskitim, ca. 85 km south of Tomsk, Western Siberia (Berezhkov 1956), requires confirmation from song recordings from the type locality. The combination *C.biguttuluseuchedickei* was restored by Willemse et al. (2009). The conspecificity of *C.yersini* sensu Bukhvalova, 1993 nec Harz, 1975 and *C.miramae* (Vorontsovsky, 1928) are absolutely correct.

Later on, Benediktov (2005) established the synonymy *C.porphyropterus = C.brunneusmistshenkoellus* Oliger, 1974 on the basis of investigation of the types of *C.brunneusmistshenkoellus* Oliger, 1974 from Tolyatti, Samara region. However oscillograms of the song of *C.maritimus* from Tolyatti (Benediktov and Mikhailenko 2017) cast doubt on this synonymy.

The status of *C.brunneus* (Thunberg, 1815) is unambiguous. In addition to the nominotypical subspecies, this species includes *C.brunneusmistshenkoellus* mentioned above and *C.brunneusbrevis* Klingstedt, 1939 from Southern Finland (Klingstedt 1939), the statuses of which require further clarification.

In the current paper, we consider the following three taxa: *C.brunneus* (Thunberg, 1815), *C.maritimus* Mistshenko, 1951, and *C.miramae* (Vorontsovsky, 1928). *C.maritimustsejensis* Bukhvalova, 1993, *C.meridionaliskarakalensis* Sytshev et Woznesenskij, 1995, *C.brunneusmistshenkoellus* Oliger, 1974, and *C.brunneusbrevis* Klingstedt, 1939 are excluded from the consideration since their statuses are unclear.

#### Chorthippus brunneus

Taxon classificationAnimaliaOrthopteraAcrididae

(Thunberg)

6FE54AA0-5782-5692-B666-E86DEB66A3BA

Gryllus brunneus Thunberg, 1815: 256.

##### Material examined.

**Bulgaria: 4** Sofia region, lake Iskyr, 29.VI.2002, 1 ♂ 5 ♀, leg. V. Vedenina, song recordings in 1 ♂ (CV); **Moldova**: **10** Vinnitza region, Volchinetz, ab. 5 km W Mogilev-Podol’sky, 17.VII.1997, 1 ♂, leg. V. Vedenina (CV); **Romania**: **11** Dobrudzha region, 14 km S Constantza, Ephoria-Nord, 09.IX.1997, 2 ♂ 3 ♀, leg. A. Loginov (ZMMU); **Ukraine: 8** Ivano-Frankovsk region, environs of Mikulichin, 09–14.VIII.1996, 6 ♂ 1 ♀, leg. V. Vedenina (CV); **9** Khmelnitsky region, 28 km NNW of Kamenetz-Podolsky, near Beloe, 25.VI.2010, 1 ♂ 1 ♀, leg. V. Vedenina, song recordings in 1 ♂ (CV); **12** Odessa region, Kiliya district, environs of Vilkovo, 30.VI.1997, 2 ♂, leg. V. Vedenina (CV); **13** Odessa region, ab. 30 km NW of Belgorod-Dnestrovsky, near Krasnaya Kosa village, 29.VI.1997, 1 ♂, leg. V. Vedenina (CV); **16** Nikolaev region, Pervomaisk district, surr. of Kuripchino village, 27.06.1997, 1♂, leg. V. Vedenina (CV); **18** Cherkassy region, Kanev district, Kanev reserve, 12–18.VI.1996, 12 ♂ 5 ♀, leg. V. Vedenina (ZMMU); **19** Kirovograd region, environs of Aleksandriya, 04.VII.1997, 2 ♂ 2 ♀, leg. V. Vedenina (CV); **20** Nikolaev region, Pervomaisk district, environs of Kuripchino village, beach of Yuzhny Bug river, 27.VI.1997, 1 ♂ 1 ♀, leg. V. Vedenina, song recordings in 2 ♂ (CV); **22** Poltava region, Mirgorod district., V.Sorochintzy, 27–28.VI.1985, 4 ♂ 5 ♀, 25–28.VII.1993, 3 ♂ 5 ♀, 24.VII–26.VIII.1994, 5 ♂, leg. V. Vedenina, song recordings in 6 ♂ (ZMMU, CV); **25** Dnipro region, Pavlograd district, Samara reserve, 12–15.VII.1996, 4 ♂ 4 ♀, leg. V. Vedenina (CV); **Russia: 1** Kaliningrad region, environs of Svetlogorsk, forest road, 16.VIII.2005, 3 ♂ 1 ♀, leg. N. Kulygina, song recordings in 1 ♂ (CV); **14** St-Peterburg, 27.08.1997, 1 ♂, leg. V. Vedenina (CV); **32** Voronezh region, Novaja Usman’ district, near Maklok village, 29.VI.2006, 3 ♂, leg. N. Kulygina (CV); **35** Kostroma region, Manturovo district, environs of. Anosovo, 07–08.VIII.2009, 2 ♂ 1 ♀, leg. V. Vedenina, song recordings in 2 ♂ (CV); **40** Saratov region, Krasny Kut district, near D‘yakovka, 17.VII.2004, 3 ♂, leg. D. Tishechkin, song recordings in 2 ♂ (ZMMU).

##### Distribution.

(Fig. 1). The range of this species extends from Europe to the south-western part of European Russia. In Europe this species occurs over a wide range, excluding the central and southern part of the Iberian Peninsula and Greece (Ragge and Reynolds 1988, Sirin et al. 2010). Further to the east, it occurs in the Baltic republics, Belarus, Moldova, and Ukraine. The eastern border of the range lies on the longitude of the Saratov and Kostroma regions of Russia. The species tends to be mesophilic. The range of *C.brunneus* overlaps with that of *C.maritimus* in south-eastern Europe, Moldova, Ukraine, and the south-eastern part of European Russia.

##### Recognition.

(Table 1, Fig. 3). The males of *C.brunneus* can be distinguished from the males of *C.miramae* and *C.maritimus* by a short stridulatory file (Fig. 3A). This, however, is not applicable to the females (Fig. 3B). Both sexes of *C.brunneus* are characterized by the lowest number of stridulatory pegs (58–93 in ♂, 51–95 in ♀.). In comparison with *C.miramae* and *C.maritimus*, both sexes of *C.brunneus* tend to have the shortest pronotum, the narrowest C & Sc areas of fore wing, and the stigma closest to the wing tip (Table 1). The PCA applied to 6 characters shows a substantial overlap between *C.brunneus* and *C.maritimus* (Fig. 3C, D). In PCA, however, we do not use the number of stridulatory pegs, since this value was measured for a small number of males. Meanwhile, it was previously shown that *C.brunneus* can be easily distinguished from all other species of the *C.biguttulus* group by the lowest number of stridulatory pegs, especially in nominate subspecies (Oliger 1974; Ragge and Reynolds 1988; Bukhvalova 1993; Willemse et al. 2009).

##### Calling song

(Table 2, Figs 4, 5). The calling song of *C.brunneus* consists of several short echemes repeated at the rate of about 0.3–2.1 /s. Each echeme lasts on average 0.1–0.4 s and has a relatively stable temporal structure. It consists of short pulses, which are grouped into 4–7 syllables (Fig. 4C). The gaps between the subsequent syllables can’t be traced by the sound analysis, but they can be distinguished by the analysis of the leg movements. The two legs are moved with a large phase shift, and sometimes almost alternately (Fig. 4E). Each leg generates one short pulse during a straight upstroke, whereas two short pulses are produced during a two-step downstroke. The pulse duration and the pulse rate vary in the ranges of 7–8 ms and 91–111/s, respectively (at the temperature 29–30°C). The population from loc. 40, shows an extremely long echeme duration and low echeme and pulse rate (Table 2). Notably, the values are relatively stable within the same population.

**Table 2.** Calling songs parameters of *Chorthippusbrunneus*. For each parameter, medians, the lower and upper quartiles are shown.

Locality	Number of recorded males (measurements)	Temperature, ˚ C	echeme duration, s	echeme rate, /s	pulse rate, /s
1	1 (10)	32	0.2	0.7	125
			0.1; 0.2	0.6; 0.9	115; 161
4	1 (9)	31–35	0.2	1.1	100
			0.2; 0.2	0.7; 1.9	100; 122
9	1 (10)	24–25	0.2	0.7	143
			0.2; 0.2	0.6; 0.8	143; 167
20	2 (16)	30	0.2	2.1	100
			0.2; 0.2	1.0; 2.5	83; 111
22	6 (51)	29	0.2	1.1	91
			0.2; 0.2	0.4; 1.3	83; 111
35	2 (20)	29–30	0.1	1.1	111
			0.1; 0.1	0.7; 2.5	91; 129
40	2 (24)	28; 32–33	0.4	0.3	57
			0.3; 0.5	0.2; 0.4	51; 66

##### Courtship and rivalry songs.

The courtship and rivalry (Fig. 4F, G) songs of *C.brunneus* are similar to the calling song.

#### Chorthippus maritimus

Taxon classificationAnimaliaOrthopteraAcrididae

Mistshenko

49E8D33F-C455-5380-BB03-57B98E5FA5F2

Chorthippus miramae Ramme, 1939: 131, nomen nudum.
Chorthippus meridionalis Mistshenko, 1950: 790.
Chorthippus biguttulus maritimus Mistshenko, 1951: 514.
Chorthippus miramae Ramme, 1951: 389.
Chorthippus biguttulus eximius Mistshenko, 1951: 515, syn. n.
Chorthippus bornhalmi Harz, 1971: 336, syn. n.
Chorthippus miramaellus Woznessenskij, 1996: 204.
Chorthippus sinuatus Mistshenko et Woznessenskij, 1996: 204.

##### Material examined.

**Bulgaria: 4** Sofia region, lake Iskyr, 29.VI.2002, 6 ♂ 5 ♀, leg. V. Vedenina (ZMMU); **5** Vraca region, ab. 3 km S of Vraca, Vracniki Balekan National Park, Memorial Botev, 30.VI.2002, 2 ♂, leg. V. Vedenina (CV); **Greece: 2** Phthiotis, environs of Timfristos, NE slope, 27.V.1998, 1 ♂, leg. V. Vedenina (CV); **3** Phthiotis, ab 40 km NW Lamia environs of Lautra Kaitsas, 26.V.1998, 3 ♂ 1 ♀, leg. V. Vedenina (CV); **6** Macedonia, Drama, Mt Falakro above Volakas, 5 km NE Elatia, 24.VII.2004, 1 ♂, leg. V. Vedenina, song recordings in 2 ♂ (CV); **7** Macedonia, Drama, W. Rodopi, 5 km NE Elatia, 23.VII.2004, 1 ♂ 1 ♀, leg. V. Vedenina (CV); **Ukraine: 15** Odessa region, near Sychavka, 03.VII.1997, 5 ♂, leg. V. Vedenina (ZMMU); **17** Kirovograd region, Novoukrainka district, environs of Pomoshnaya, 26.VI.1997, 2 ♂, leg. V. Vedenina, song recordings in 2 ♂ (CV); **21** Kherson region, Chernomorsky nature reserve, Solyonoozerny area, 25.VII–05.VIII.1995, 2 ♂ 1 ♀, leg. V. Vedenina (CV); **23** Crimea, Bakhchisaray district, 3–4 km E of Gluboky Yar, 11.VI.1997, 4 ♂, leg. D. Tishechkin, song recordings in 4 ♂ (ZMMU); **24** Crimea, Simferopol’ district, environs of Pereval’noe, 20.VI.1997, 3 ♂, leg. D. Tishechkin, song recordings in 3 ♂ (ZMMU); **25** Dnipro region, Pavlograd district, Samara reserve, 12–15.VII.1996, 6 ♂, leg. V. Vedenina (CV); **26** Crimea, Kerch peninsula, E shore of Kazantip bay, environs of cape Chagany, 26.VI.1997, 1 ♂, leg. D. Tishechkin, song recordings in 1 ♂ (ZMMU); **27** Kharkov region, Izjum district, Kamyshevacha, 15.VII.1996, 5 ♂ 1 ♀, leg. V. Vedenina (ZMMU); **28** Kharkov region, Izjum, Kremenetz hill, 15.VII.1996, 1 ♂, leg. V. Vedenina (CV); **Abkhazia: 34** Sukhumi region, slopes near highway Sukhumi – Gagra, 21–22.X.2005, 5 ♂ 5 ♀, leg. V. Vedenina, song recordings in 3 ♂ (ZMMU); **Russia**: **33** Krasnodarsky krai, near highway Krasnaya Poljana – Adler, 22.X.2005, 4 ♂ 3 ♀, leg. V. Vedenina, song recordings in 4 ♂ (CV); **39** Saratov, slopes near Polivanovka, 28.VI.2020, 2 ♂, leg. V. Vedenina, song recordings in 2 ♂ (CV); **41** Saratov region, Krasnokutsk district, near D’yakovka, 28.VI.2020, 6 ♂ 1 ♀, leg., song recordings in 5 ♂ (CV); **43** Saratov region, SW from Khvalynsk, environs of Ul’yanino village, 19.VII.2005, 3 ♂, leg. D. Tishechkin, song recordings in 3 ♂ (ZMMU); **44** Saratov region, ab. 6 km NW of Ershov, 22.VI.2018, 3 ♂, leg. V. Vedenina (CV); **45** Saratov region, 15 km NE Ozinki, 23.VI.1996, 4 ♂, leg. D. Tishechkin, song recordings in 4 ♂ (ZMMU); **42** Krasnoyarsk region, Astrakhan‘ district, environs of Dosang railway station, 03.VII.2000, 1 ♂, leg. D. Tishechkin, song recordings in 1 ♂ (ZMMU); **75** Irkutsk region, Olkhon district, 20 km from Jelantsy to strait Olkhonskie vorota, 15.VII.2003, 4 ♂, leg. D. Tishechkin, song recordings in 4 ♂ (ZMMU); **77** Buryatia, Barguzin valley, Ina river, 4 – 5 km downstream from Ina, 17.VII.2007, 3 ♂, leg. D. Tishechkin, song recordings in 2 ♂ (ZMMU); **78** Chita region, Klichka range, ab. 15 km W Klichka, 22.VII.2003, 2 ♂, leg. D. Tishechkin, song recordings in 1 ♂ (ZMMU); **79** Amur region, 15 km S Svobodny, environs of Malaya Sazanka, 05.VII.1995, 4 ♂, leg. D. Tishechkin, song recordings in 4 ♂ (ZMMU); **80** Primorskiy kray, Pogranichny district, environs of Barabash-Levada, 20.VII.1995, 3 ♂, leg. D. Tishechkin, song recordings in 3 ♂ (ZMMU); **81** Primorskiy kray, Pogranichny district, Khanka lake, 15 km S Turiy Rog, 21.VII.2006, 3 ♂, leg. D. Tishechkin, song recordings in 3 ♂ (ZMMU); **82** Southern Sakhalin, environs of Sokol, 02.VIII.2015, 4 ♂, leg. D. Tishechkin, song recordings in 3 ♂ (ZMMU); **Kazakhstan: 62** Almaty region, 40 km N from Almaty, environs of Kara-Oi village, 12.VI.2017, 1 ♂, leg. D. Tishechkin, song recordings in 1 ♂ (ZMMU); **63** Almaty, botanical garden, 07.VII.1994, 3 ♂, leg. D. Tishechkin, song recordings in 3 ♂ (ZMMU); **65** Almaty region, ab. 20 km NE of Taldykorgan, 02.VII.2016, 4 ♂, 1 ♀, leg. V. Vedenina & T. Pushkar, song recordings in 1 ♂ (CV); **66** Kazakhstan, Almaty region, near Kapal, 01.VII.2016, 1 ♂, leg. V. Vedenina & T. Pushkar, song recordings in 1 ♂ (CV); **67** Kazakhstan, Almaty region, ab. 2.5 km W of Kapal, 02.VII.2016, 4 ♂ 4 ♀, leg. V. Vedenina & T. Pushkar, song recordings in 2 ♂ (ZMMU); **68** Urzhar region, 27 km SSE Taskesken, 5.5 km NW Karakol, 24.VI.2019, 1 ♂, leg. D. Tishechkin, song recordings in 1 ♂ (ZMMU); **Turkmenistan**: **49** Ahal region, Kaka district, 6–7 km S of Dushak, 14.V.2014, 3 ♂, leg. D. Tishechkin, song recordings in 3 ♂ (ZMMU); **Kyrgyzstan**: **51** Batken region, Leilek district, Turkestan range, 12 km S from Katran village, 11.VII.2014, 1 ♂, leg. D. Tishechkin, song recordings in 1 ♂ (ZMMU); **53** Batken region, N shore of Tortkul’skoye reservoir, 12 km WSW Batken, 09.VII.2014, 1 ♂, leg. D. Tishechkin, song recordings in 1 ♂ (ZMMU); **54** Jalal-Abad region, Chatkal range, Sary-Chelek nature reserve, environs of Arkyt, 22.VII.2008, 2 ♂, leg. D. Tishechkin, song recordings in 1 ♂ (ZMMU); **57** Chuy region, Jayyl district, Karakol river, 10 km upstream from confluence with Suusamyr, 07.VII.2016, 1 ♂, leg. D. Tishechkin, song recordings in 1 ♂ (ZMMU); **58** Chuy region, Djumgal river, between Baizak and Chaek, 30.VI.2014, 1 ♂, leg. D. Tishechkin, song recordings in 1 ♂ (ZMMU); **64** Issyk-Kul‘ region, Tossor river, 18 km E from Kadji-Sai, 15.VII.2013, 1 ♂, leg. D. Tishechkin, song recordings in 1 ♂ (ZMMU).

##### Distribution.

(Fig. 1). *C.maritimus* is a widespread trans-Palearctic species. It includes *C.bornhalmi* from the Balkans and Anatolia (Willemse et al. 2009; Sirin et al. 2010; Skejo et al. 2018) and as *C.biguttuluseximius* from Sukhumi, Abkhazia (Mistshenko 1901). It also occurs in Moldova and southern Ukraine (Heller et al. 1998). In the territory of Russia, its range stretches from Krasnodarsky krai to Sakhalin along the southern border. This species also occurs in Caucasus, southern Kazakhstan, Turkmenistan, very likely Uzbekistan, Kyrgyzstan, Mongolia, northern-east China, Korea and Japan (Storozhenko 2002). The ranges of *C.maritimus* and *C.brunneus* overlap in Eastern Europe, Ukraine and the south-eastern part of European Russia. Moreover, *C.maritimus* and *C.brunneus* often occur syntopically. The range of *C.maritimus* also overlaps with the range of *C.miramae* in the south-eastern part of European Russia and in surroundings of the Baikal Lake, however, they do not occur in the same biotopes.

##### Recognition.

(Table 1, Fig. 3). The males of *C.maritimus* can be distinguished from the males of *C.brunneus* by the longer stridulatory file (Fig. 3A) and the higher number of stridulatory pegs (see Description). These characters are also mentioned as the distinguishing features between *C.brunneus* and *C.bornhalmi* by other authors (Willemse et al. 2009; Skejo and Ivcovic 2015). The length of stridulatory file in *C.maritimus* is intermediate between those in *C.miramae* and *C.brunneus*. Both sexes of *C.maritimus* also tend to have the longest fore wings and pronotum in comparison with *C.miramae* and *C.brunneus* (Table 1). *C.maritimus* can be also distinguished from other species of the *biguttulus* group by the narrower costal area of fore wing. By contrast, *C.maritimus* differs from *C.mollis* by the wider costal area of fore wing and by the lower density of stridulatory pegs (Bukhvalova 1993; Oliger 1974). *C.bornhalmi* and *C.biguttuluseximius* are not different in morphology from *C.maritimus* from Ukraine and Russia.

##### Description.

(Table 1, Fig. 3). The head structure as in genus. Ratio length of vertical diameter of eye to maximum length of foveolae 2.8–3.4 in ♂, 3.0–3.2 in ♀; ratio minimum interocular distance to length of subocular groove 0.6–0.8 in ♂, 0.7–0.9 in ♀. Antennae filiform. Prozona is slightly shorter than metazona; median carina is distinct and continuous. Lateral pronotal keels are distinctly incurved, ratio between minimum and maximum widths 2.3–2.6 in ♂, 2.3–2.9 in ♀. In western populations keels are more angled, min/max width ratio up to 3.0. Tympanal aperture slit-like, 2.3–2.8 times in ♂, 2.6–2.8 in ♀ as long as broad. Fore and hind wings well developed in both sexes, wings far surpassing the apices of the hind knee. Costal area of fore wing has maximum width in the middle part or in the last third of the wing. Subcostal area narrow, its width 0.25–0.3 mm in ♂, 0.15–0.2 mm in ♀ (measured on the line of maximal width of costal area). Ratio width of fore wing to C & Sc areas 3.1–3.5 in ♂, 4.4–4.7 in ♀. Apical constriction (distance from C and Sc confluence to the wing tip) prolonged, ratio length of apical constriction to the wing length 3.3–3.8 in ♂, 3.5–3.8 in ♀. Stigma far from the wing tip, ratio length between stigma center and the wing tip to the wing length 2.4–2.7 in ♂, 2.3–2.5 in ♀. Hind femur gracile, ratio femur length to maximum width 4.4–4.6 in ♂, 4.4–4.7 in ♀. Stridulatory file consists of one row, its length nearly equal to the distance between last peg and tip of hind knee. The number of stridulatory pegs 100–168 in ♂, 104–157 in ♀. Body coloration varies from light straw to dark brown, sometimes with a red tone. The ventral side of the body lighter than dorsal side, and densely pubescent. Fore wings smoky, with a few dark spots in M area. Hind wings transparent at the base and slightly smoky in apical part, distal half of C area smoky or brownish. Hind femur in the inner side with black lengthwise line. Hind knees dark brown or blackish, particularly on upper lobe. Hind tibiae orange or reddish.

Measurements in mm. Body length: 15–18 in ♂, 19–26 in ♀, pronotum length: 3.1–3.4 in ♂, 4.1–4.4 in ♀, fore wing length: 14.1–15.5 in ♂, in 17.2–18.5 in ♀, fore wing width 3.1–3.4 in ♂, 3.2–3.5 in ♀, hind femur length: 9.8–10.6 in ♂, 12.8–14.1 in ♀.

##### Calling song

(Table 3, Figs 5, 6). The calling song of *C.maritimus* usually contains one to several echemes of median duration ranged from 1 to 4 s. In some populations (49, 62, 63), however, the median echeme duration is higher, ranging between 5–11.1 s (Table 3, Fig. 5C). The echeme rate also greatly varies between different populations (0.05–0.42 / s). The number of syllables per echeme varies in the range of 15 to 40, in populations with prolonged echemes – in the range from 40 to 70. The syllable duration is relatively stable within the same population; however, its median duration can vary between the populations in the range of 86–162 ms (Fig. 5D). At the beginning of each echeme, the sound is very soft, but then it reaches maximum loudness after the first third of the echeme duration, being constant until the echeme end (Fig. 6D). The syllables are generated by the leg movements with a small phase shift, which comprise the straight upstroke and stepwise downstroke (Fig. 6E, F). Both upstroke and downstroke have the similar duration. The leg upstroke generates a noisy sound with unclear structure and slightly increasing amplitude; the stepwise downstroke generates 4–5 distinct pulses. The pulses, however, can be sometimes fuzzy. The durations and rates of echeme and syllable in *C.bornhalmi* (from loc. 6) and in *C.biguttuluseximius* (from loc. 34) fall into the range of values in *C.maritimus* from several localities (Table 3, Fig. 5C, D). The syllable structure is also quite similar in *C.bornhalmi* (Fig. 6E) and *C.biguttuluseximius* (Fig. 6F).

**Table 3.** Calling songs parameters of *Chorthippusmaritimus*. For each parameter, medians, the lower and upper quartiles are shown.

Locality	Number of recorded males (measurements)	Temperature, ˚ C	echeme duration, s	echeme rate, /s	syllable duration, ms	syllable rate, /s
6	2 (10)	30	4.0	0.19	103	8.5
			3.6; 4.6	0.18; 0.19	99; 105	8.3; 9.1
17	2 (10)	32	1.0	0.20	129.5	9.4
			0.9; 1.1	0.18; 0.25	127; 132	8.6; 10.0
23	4 (40)	31–35	1.7	0.25	102	9.3
			1.5; 1.9	0.20; 4.5	96; 106	8.9; 9.5
24	3 (18)	24–25	1.4	0.3	104	8.8
			1.2; 1.6	0.29; 0.34	102; 112	8.5; 9.0
34	3 (14)	30	2.8	0.42	136	7.0
			2.0; 4.4	0.21; 0.46	119; 159	5.6; 8.1
39	2 (13)	29	2.1	0.24	103	9.2
			1.4; 2.7	0.22; 0.27	100; 106	9.0; 9.4
41	5 (15)	29–30	2.4	0.20	100	9.3
			2.0; 2.9	0.16; 0.22	95; 108	8.7; 9.9
43	3 (12)	28; 32–33	1.3	0.25	86	10.1
			1.2; 1.7	0.22; 0.28	81; 124	7.2; 10.8
45	4 (12)	32–36	2.0	0.23	119	7.8
			1.6; 2.3	0.19; 0.24	110; 127	7.2; 8.1
49	3 (25)	34–35	10.7	0.07	159	6.4
			4.9; 12.2	0.06; 0.08	157; 165	6.3; 6.5
54	1 (10)	35–39	3.2	0.16	135	7.0
			2.0; 5.2	0.13; 0.26	133; 136	6.9; 7.0
62, 63	4 (11)	30–32; 35	11.1	0.05	162	5.9
			7.8; 11.5	0.04; 0.05	134; 164	5.7; 6.1
75	4 (12)	31	2.2	0.14	86	10.1
			1.7; 5.2	0.13; 0.18	83; 94	9.7; 10.5
77	2 (15)	20; 27–30	2.5	0.13	133	7.0
			1.7; 5.2	0.12; 0.23	124; 147	6.3; 7.7
79	4 (13)	31	2.0	0.14	90	10.3
			1.7; 2.9	0.09; 0.18	87; 104	9.2; 10.7
80	3 (18)	38–40	2.1	0.13	87	10.9
			1.9; 2.5	0.07; 0.16	85; 90	9.6; 11.1
82	3 (20)	35–40	2.3	0.15	88	10.5
			1.8; 3.5	0.12; 0.18	85; 90	10.3; 11.2

##### Courtship song.

The courtship song of *C.maritimus* is similar to the calling song.

##### Rivalry song

(Fig. 6G, H). The rivalry song of *C.maritimus* contains echemes of a shorter duration than the calling song. In some males the first syllable of the rivalry echeme lasts 1.5–2 times as long as the subsequent syllables, which results from the prolonged first downstroke (Fig. 6H). The pulses produced during the first downstroke are repeated twice as slowly as the pulses of the subsequent syllables. The subsequent 2–8 syllables are of the same structure as the syllables in the calling song.

#### Chorthippus miramae

Taxon classificationAnimaliaOrthopteraAcrididae

(Vorontsovsky)

E12C84A5-C31F-58C5-AF46-1C78B5A7213B

Stauroderus miramae Vorontsovsky, 1928a: 12.
Stauroderus mollis porphyroptera Vorontsovsky, 1928b: 31, 34.
Chorthippus porphyropterus (Vorontsovsky, 1928): Benediktov, 1999: 42.

##### Material examined.

**Russia: 29** Krasnodarsky kray, environs of Gelendzhik, 06.X.2011, 8 ♂ 4 ♀, leg. V. Vedenina & L. Shestakov, song recordings in 3 ♂(ZMMU); **30** Krasnodarsky kray, Gelendzhik district, environs of Aderbievka, 07.VII.1997, 8 ♂ 8 ♀, leg. D. Tishechkin, song recordings in 4 ♂ (ZMMU); **31** Krasnodarsky kray, Gelendzhik district, environs of Praskoveevka; 12.VII.1997, 2 ♂, leg. D. Tishechkin (ZMMU); **36** N. Caucasus, N. Ossetia, environs of Alagir, Ardon river floodplain, 09.VIII.1990, 2 ♂ 2 ♀, leg. M. Bukhvalova, song recordings in 2 ♂ (ZMMU); **37** N. Caucasus, N. Ossetia, Sunzhensky range, environs of Elkhotovo, 10–12.VIII.1990, 2 ♂ 1 ♀, leg. M. Bukhvalova (ZMMU); **38** N. Caucasus, N. Ossetia, Sunzhensky range, environs of Bekan lake, 14.VIII.1985, 3 ♂ 3 ♀, leg. D. Tishechkin (ZMMU); **47** Orenburg region, environs of Studentzy, 14.VII.2012, 1 ♂, leg. V. Vedenina & L. Shestakov, song recordings in 1 ♂ (CV); **48** Orenburg region, environs of Guberlya railway station, 07–09.VII.1996, 37 ♂ 13 ♀, leg. D. Tishechkin, song recordings in 5 ♂ (ZMMU), 29.VI.2018, 1 ♂, leg. V. Vedenina & N. Sevastianov, song recordings in 1 ♂ (CV); **69** Altai Republic, ab. 26 km SE of Ongudai, environs of Kupchegen’, 08.VIII.2017, 5 ♂ 3 ♀, leg. V. Vedenina & N. Sevastianov, song recordings in 1 ♂ (ZMMU); **70** Tyva republic, environs of Erzin, Tore-Kchan’ lake, 31.VII.1989, 1 ♂ 1 ♀, leg. S. Byzov (ZMMU); **71** Tyva republic, environs of Erzin, Erzin river floodplain, 20.VII–06.VIII.1989, 3 ♂ 3 ♀, leg. M. Bukhvalova, song recordings in 3 ♂ (ZMMU); **72** Tyva republic, environs of Erzin, Tes-Kchem river floodplain, 03–06.VIII.1989, 3 ♂, leg. M. Bukhvalova (ZMMU); **73** Irkutsk region, Nizhneudinsk district, Uk river estuary, confluence with Uda, 02.VII.2003, 5 ♂, leg. D. Tishechkin, song recordings in 5 ♂ (ZMMU); **74** Buryatia, Selenginsk district, 5 km N from Novoselenginsk, Selenga river valley, 07.VII.2007, 5 ♂, leg. D. Tishechkin, song recordings in 5 ♂ (ZMMU); **76** Buryatia, Zaigrayevo district, 10 km Onokhoy, Bryanka river valley, 21.VII.2007, 3 ♂, leg. D. Tishechkin, song recordings in 3 ♂ (ZMMU); **Kazakhstan**: **46** West-Kazakhstan region, ab. 50 km W of Ural’sk, environs of Kamenka, 23.VI.2018, 5 ♂, leg. V. Vedenina & N. Sevastianov (ZMMU); **50** Kostanay region, Naurzum nature reserve, 04–11.VIII.1938, 13 ♂ 6 ♀, leg. Derevitskaya, 11.VIII–25.IX.1939, 3 ♂ leg. Pokrovskyi, 24.VII.1947, 1 ♂ A. Formozov (ZMMU); **52** Akmola region, Tselinograd district, ab. 4 km SWW from Zhaynak, 09.VII.2019, 3 ♂, leg. V. Vedenina, N. Sevastianov & T. Tarasova, song recordings in 1 ♂ (CV); **55** Akmola region, Arshaly district, 7 km N Vishnevka, Ishym river floodplain, 11.VII.2019, 3 ♂, leg. V. Vedenina, N. Sevastianov & T. Tarasova, song recordings in 2 ♂ (CV); **56** Akmola region, Jerementau district, 4.5 km NE from Baysary, 03.VII.2019, 2 ♂, leg. V. Vedenina, N. Sevastianov & T. Tarasova, song recordings in 2 ♂ (CV); **59** Pavlodar region, Ekibastuz district, ab. 3 km W of Schidert, 04.VII.2019, 6 ♂ 1 ♀, leg. V. Vedenina, N. Sevastianov & T. Tarasova, song recordings in 3 ♂ (ZMMU); **60** Pavlodar region, Zhelezinsky district, near Pyatiryzhsk, 22.VII 1 ♂ 1 ♀ leg. Ingenitskyi (ZMMU), 05.VII.2019, 2 ♂, leg. V. Vedenina, N. Sevastianov & T. Tarasova, song recordings in 2 ♂ (CV); **61** Pavlodar region, Terenkol’ district, bank of the Irtysh river, 05.VII.2019, 6 ♂, leg. V. Vedenina, N. Sevastianov & T. Tarasova, song recordings in 1 ♂ (CV).

##### Distribution.

(Fig. 1). The range of this species stretches in the form of a ribbon from the Black Sea coast eastwards to Transbaikalia. *C.miramae* occurs in Krasnodarsky krai and Caucasus, Orenburg region, northern Kazakhstan, Altai, Tyva, Irkutsk region and Transbaikalia. The ranges of *C.miramae* and *C.maritimus* overlap in the south-eastern part of European Russia and in surroundings of Baikal Lake.

##### Recognition.

(Table 1, Figs 2, 3). *C.miramae* can be distinguished from most species of the *biguttulus* group by remarkably long stridulatory file (Fig. 2C). This feature was previously shown by Benediktov (1999), who described the last distal stridulatory peg to be situated at least at a level of the second tibial spine when tibia is attached to femur. Within the *biguttulus* group, a similarly long file is only shown in *C.biguttuluseuhedickei* von Helversen, 1989, that occurs in the southern Balkans and Anatolia and in *C.maroccanus* Nadig, 1986, that occurs in North Africa (Ragge and Reynolds 1988; Willemse et al. 2009). The latter two taxa, however, are quite different from *C.miramae* in other morphological characters and songs. In other species of the *biguttulus* group, the length of stridulatory file is noticeably shorter, and the last distal stridulatory peg is situated at least at the level of the 4^th^ tibial spine when the legs are bent (Benediktov 1999). Notably, in *C.miramae*, the number of stridulatory pegs is only slightly higher than in *C.maritimus*, and can’t be considered as a good character. *C.miramae* tends to have the longest distance between stigma and the wing tip, and the broadest width of C & Sc areas in comparison to *C.maritimus* and *C.brunneus*. The PCA based on 6 morphological characters shows that *C.miramae* represents a separate cluster from *C.maritimus* and *C.brunneus*, but it is stronger in males than in females (Fig. 3C, D).

##### Description.

(Table 1, Figs 2, 3). The head structure as in genus. Ratio length of vertical diameter of eye to maximum length of foveolae 3.2–3.6 in ♂, 2.8–3.2 in ♀; ratio minimum interocular distance to length of subocular groove 0.6–0.8 in ♂, 0.7–1.0 in ♀. Antennae filiform. Median carina distinct and continuous. Prozona slightly shorter than metazona. Lateral pronotal keels distinctly incurved, ratio minimum to maximum widths 2.1–2.6 in ♂, 2.4–2.6 in ♀. Tympanal aperture 2.8–3.3 times in ♂, 2.8–3.4 in ♀ as long as broad. Fore and hind wings well developed in both sexes, wings far surpassing the apices of the hind knee. Width of costal area of fore wing reaches its maximum in the middle or in the last third part (Fig. 2A, B). Width of subcostal area 0.3–0.35 mm in ♂, 0.2–0.23 mm in ♀ (measured along the line of maximal width of costal area). Ratio width of fore wing to width of C & Sc areas 3.0–3.2 in ♂, 4.3–4.5 in ♀. Length of apical constriction (distance from C and Sc confluence to the wing tip) is a quarter of the wing length. Ratio length between stigma center and the wing tip to the wing length 2.1–2.8 in ♂, 1.8–1.9 in ♀. Hind femur gracile, ratio femur length to maximum width 4.5–4.9 in ♂, 4.6–4.9 in ♀. Stridulatory file remarkably long in both sexes: distance between the last peg and the knee tip 2–2.7 times in ♂, 1.7–2.4 in ♀ as large as length of stridulatory file. In males, stridulatory pegs form one row and have different density along the file (Fig. 2C). Most proximal part of stridulatory file starts with several rare and dispersed pegs that are followed by more densely disposed pegs. The second part of stridulatory file more prolonged, consisting of more rare pegs with stable inter-peg intervals. In the third, most distal part the peg density decreases proportionally to the length of stridulatory file, and the pegs often do not lay in one raw. In females, stridulatory pegs arranged in one row and distributed rarer than in males. The peg density decreases from the proximal towards the distal parts. The number of stridulatory pegs 118–182 in ♂, 98–157 in ♀. Body coloration similar to coloration of *C.maritimus*.

Measurements in mm. Body length: 14–18 in ♂, 18–24 in ♀, pronotum length: 2.9–3.3 in ♂, 3.8–4.4 in ♀, fore wing length: 13.3–14.6 in ♂, in 16.4–18.3 in ♀, fore wing width 3.1–3.6 in ♂, 3.2–3.5 in ♀, hind femur length: 9.7–10.4 in ♂, 12.6–14.0 in ♀.

##### Calling song.

**Figure 7.**
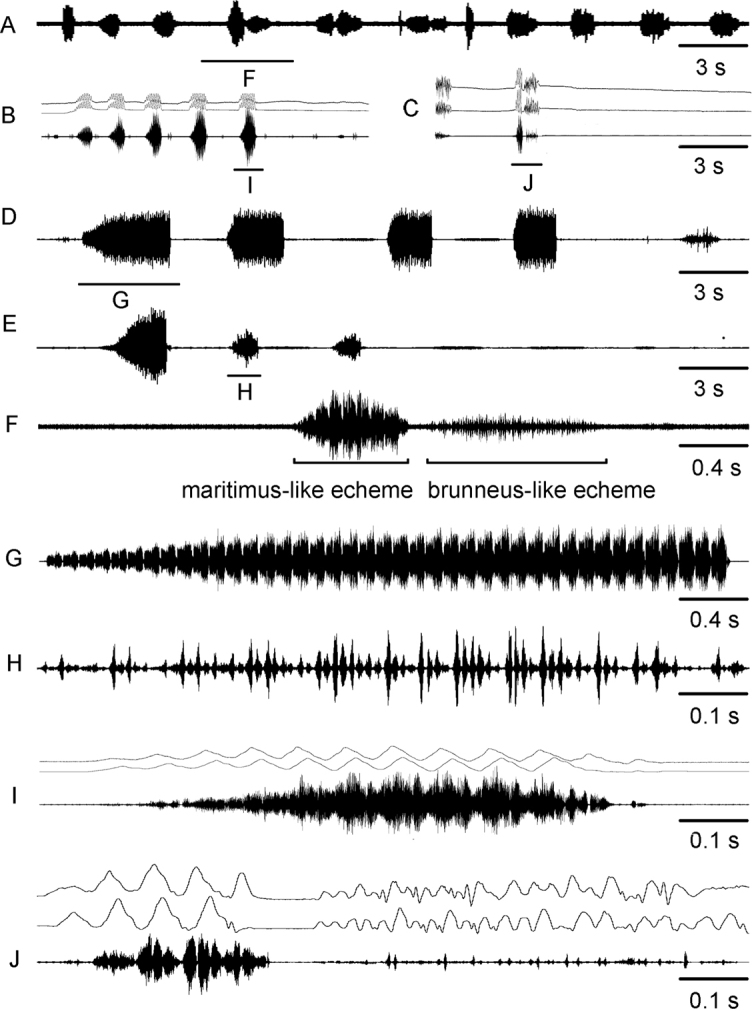
Oscillograms of calling songs of *Chorthippusmiramae* from Orenburg region **A**, West-Kazakhstan region **C** and Buryatia **D,E**. Song recordings are presented at three different speeds (faster oscillograms of the indicated parts of the songs shown in **F–J**. At small scales (**A–D**) the *maritimus*-like echemes can be distinguished from the *brunneus*-like echemes by the higher amplitude. In all oscillograms the two upper lines are recordings of hind leg movements and the lower line is the sound recording. The ambient temperature near a singing male was 34 – 35 °C in (**A,E**) and 29 – 31°C in other cases.

(Table 4, Figs 5, 7). The calling song of *C.miramae* includes the two types of randomly alternating echemes, typical *maritimus*-like and optional *brunneus*-like echemes. The first echeme type was present in the songs of all 34 males recorded, the second echeme type – in the songs of 28 males. The song usually starts with the *maritimus*-like echeme, which is similar to the *C.maritimus* calling song, but lasting shorter (the median duration varies in the range of 0.3–2.9 s). The number of syllables per echeme varies in the range of 5 to 35. Each echeme starts with the low-amplitude syllables. In short echemes, the amplitude reaches its maximum in about the echeme middle (Fig. 7F). In long echemes, the amplitude gradually increases, and keeps a constant level after about one quarter of an echeme (Fig. 7G). The syllables are about 1.5 times as short as the syllables in *C.maritimus*, lasting in the range of about 66–114 ms (Table 4). The syllable duration is rather stable within one population; however, it is more variable between populations. Oscillographic analysis shows no distinct pulses within the syllables in some populations, whereas distinct pulses are visible on the oscillograms of the songs from other populations. The shift between the two legs is greater in *C.miramae* than in *C.maritimus* (Fig. 7I, J).

**Table 4.** Calling songs parameters of *Chorthippusmiramae*. For each parameter, medians, the lower and upper quartiles are shown.

Locality	Number of recorded males (measurements)	Temperature, ˚ C	*maritimus*-like part				*brunneus*-like part		
			echeme duration, s	echeme rate, /s	syllable duration, ms	syllable rate, /s	echeme duration, s	echeme rate, /s	pulse rate, /s
29–30	7 (40)	25–28;	2.9	0.28	114	7.81	1.4	0.19	76.9
		30–32	1.8; 3.8	0.16; 0.29	99;135	7.16;8.53	1.0; 1.7	0.14; 0.20	66.7; 90.1
36	2 (20)	30	2.4	0.07	86	9.2	0.8	0.21	95.5
			2.2; 2.7	0.07; 0.07	78; 98	9.7; 11.3	0.8; 0.9	0.17; 0.22	81.7;102.8
48	6 (60)	28–30;	0.9	0.47	66	13.8	0.4	0.31	83.3
		34	0.8; 0.9	0.40; 0.59	60; 74	12.5; 14.9	0.3; 0.8	0.29; 0.37	71.4; 100
56	2 (15)	31	0.3	1.4	69	14.6	n/a	n/a	n/a
			0.3; 0.4	1.2; 1.8	61; 76	13.2; 16.3			
71	3 (13)	25–26;	0.8	0.40	76	10.5	1.0	n/a	47.6
		30	0.8; 1.1	0.36; 0.45	64; 91	9.6; 13.0	0.9; 1.2		23.4; 62.5
73	5 (26)		0.8	0.36	68	13.0	0.3	0.31	83.3
			0.7; 0.8	0.34; 0.44	64; 75	12.4; 14.3	0.3; 0.7	0.30; 0.33	70.2; 93.2
74	5 (20)	29–30	2.0	0.16	85	10.7	1.2	0.26	76.9
			2.0; 2.8	0.15; 0.17	81; 91	10.1; 11.2	1.0; 1.3	0.22; 0.28	71.4; 83.3
76	3 (10)	35	2.6	0.20	80	11.0	1.3	0.23	83.3
			1.9; 3.0	0.19; 0.21	75; 85	10.7; 11.6	1.0; 1.5	0.20; 0.30	71.4; 90.9

*n/a – non-applicable

The *brunneus*-like echemes are more often produced by the males from the Siberian and the east-european Russian populations, but they are rare in the songs from northern Kazakhstan. The echeme duration in *C.miramae* is almost three times as high as in *C.brunneus* (Fig. 5A). Similarly to *C.brunneus*, the *C.miramae* echeme consists of the short pulses, the amplitude of which gradually increases, reaching maximum intensity at about half of its duration, and then gradually decreases towards the end. The pulse duration and the pulse rate in *C.miramae* are almost the same as in *C.brunneus* (9–13 ms and 77–96 /s respectively, data are given for 29–30°C). However, the leg movement patterns are different in two species. In *C.miramae*, the *brunneus*-like echeme is produced by simple up and down leg-movements that vary in amplitude and duration (Fig. 7J). In *C.brunneus*, each leg generates a simple upstroke but a two-step downstroke (Fig. 4D). The oscillographic analysis of the *C.miramae* song shows that the pulses highly vary in amplitude and duration, whereas the pulses in the *C.brunneus* song are much more stable in these parameters. In some males of *C.miramae*, the pulses are tended to group into syllables; the pulse number per syllable is unstable (Fig. 7H).

The order of the two echeme types in the *C.miramae* song is erratic, though there are some common variants in different populations. For example, several *maritimus*-like echemes are followed by one *brunneus*-like echeme (Fig. 7D). Another variant implies alternation of the two echeme types. A rarer case is when one *maritimus*-like echeme is followed by several echemes of the second type (Fig. 7A, E). The intervals between echemes of the same type may exceed the echeme duration 1.5–3 times for the *maritimus*-like echemes, and 3–5 times for the *brunneus*-like echemes. An interval between the *maritimus*-like and the subsequent *brunneus*-like echemes can be very short (Fig. 7F, J), or can exceed the echeme duration 3–5 times.

##### Courtship song and female response song.

**Figure 8.**
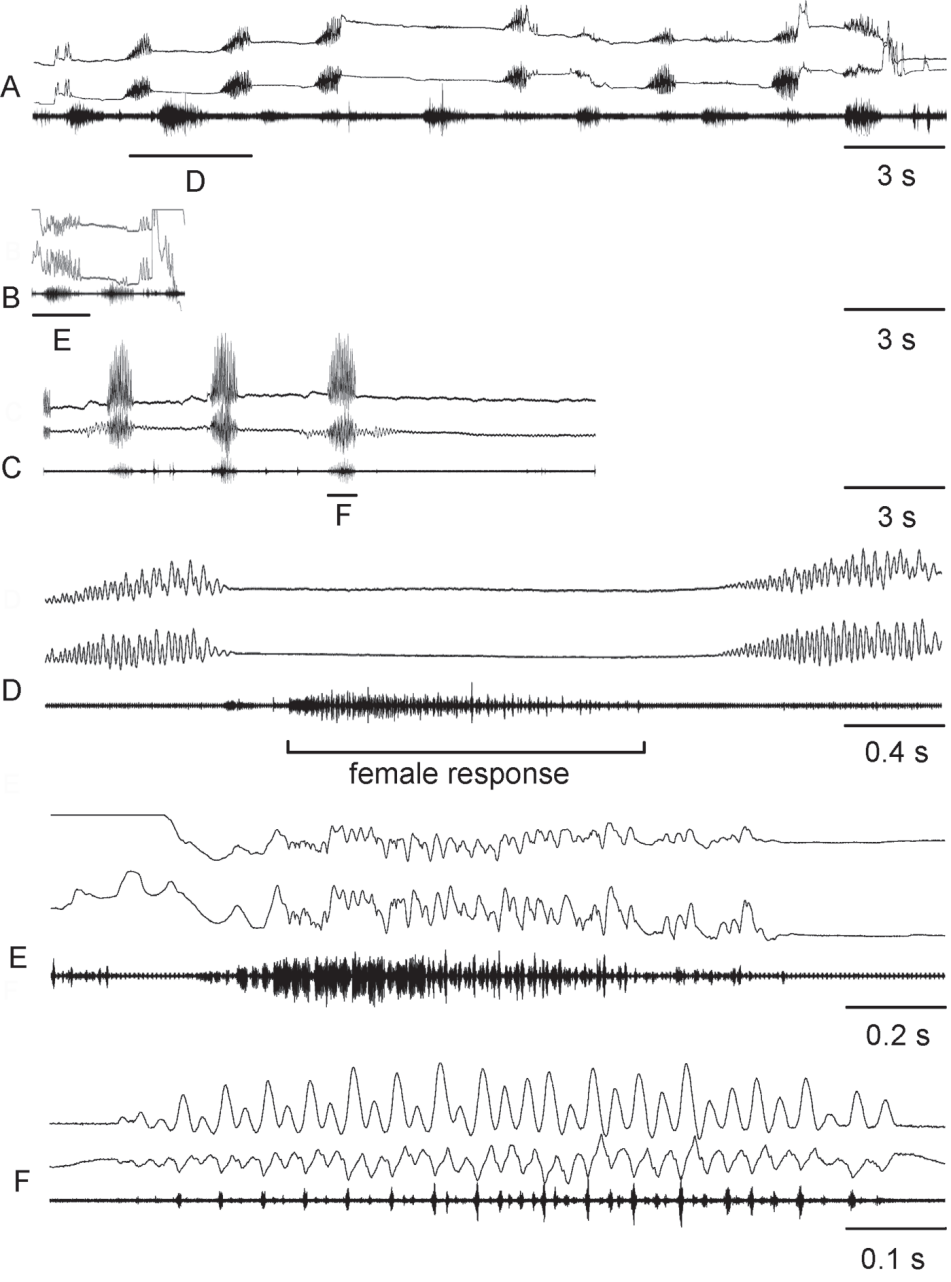
Oscillograms of courtship songs and female response songs in *Chorthippusmiramae* from Pavlodar region **A** West-Kazakhstan region **B** and Altai republic **C**. Song recordings are presented at three different speeds (faster oscillograms of the indicated parts of the songs shown in **D–F**). During courtship, a male can produce audible (**C,F**) or silent (**A,D**) variants of song. Female responses with leg movements recordings (B, E) and without them (**A,D**) are shown. In all oscillograms the two upper lines are recordings of hind leg movements and the lower line is the sound recording. The ambient temperature near a singing specimen was 29 – 31°C.

(Fig. 8). The courtship song of *C.miramae* consists of the *brunneus*-like echemes. However, the courtship sound is much softer than in the calling song. The courtship echemes are shorter than in the calling song, not reaching 1 s (the median duration is about 0.4 s). The echemes are usually repeated at the rate of about 0.2–0.6/s, and their duration varies from 0.7 to 1.0 s. Pulses are short (6–9 ms), frequent (repeated at the rate of 61–95/s), and of a low amplitude (Fig. 8F). In some cases, the leg movements do not produce any sound at all (Fig. 8A, D).

A female produces the *brunneus*-like song in response to the male courtship or rivalry song (Fig. 8A, B). The female alternates her response echemes with the male echemes (Fig. 8D). The duration of the female echeme is similar to that in the male courtship, or 1.5–2 times longer than in the male courtship. The leg movement pattern in the female response song is similar to that in the male courtship song, but less regular (Fig. 8E, F). The pulses are longer (10–21 ms) and repeated at the rate of 43–77/s, especially in the first third of the echeme (Fig. 8D, E).

##### Rivalry song.

**Figure 9.**
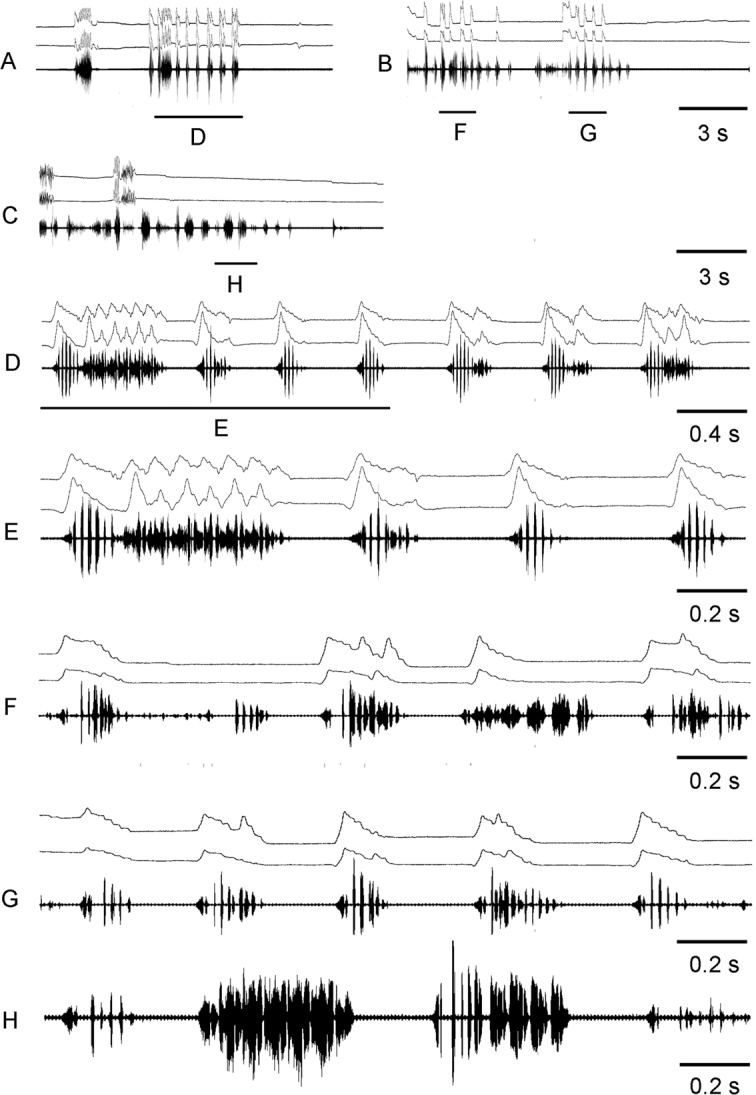
Oscillograms of rivalry songs in *Chorthippusmiramae* from Altai republic **A** and Pavlodar region of Kazakhstan **B,C**. Song recordings are presented at three different speeds (faster oscillograms of the indicated parts of the songs shown in **D–H**). In all oscillograms the two upper lines are recordings of hind leg movements and the lower line is the sound recording. The ambient temperature near a singing male was 29 – 31°C.

(Fig. 9). Several males of *C.miramae* sitting close to each other produce a diversity of echemes of different duration, structure and leg movement pattern. For example, one can find a rivalry song similar to that of *C.maritimus*, which starts with the prolonged first syllable, which results from the prolonged first downstroke (Fig. 9D, E). The pulses produced during the first downstroke follow twice as slowly as the pulses of the subsequent syllables. The subsequent syllables are of the same structure as in the *maritimus*-like echeme of the calling song.

Most often, the males produce single syllables similar to the first one with distinct pulses described above. These syllables are repeated at the rate of about 2–2.5 /s (Fig. 9F, G). Notably, the two legs may produce different number of the up and down strokes. Rarely, the males produce the *maritimus*-like echeme without the first syllable of distinct pulses (Fig. 9H).

The same male may produce echemes of different structure in the rivalry situations. Some females are actively responding to the male rivalry songs.

## Discussion

### What is the function of the long stridulatory file?

The morphological analysis conducted in the current study shows that one character, the length of stridulatory file, appears to be the most reliable character to distinguish *C.miramae*, *C.maritimus* and *C.brunneus*. The difference in the file length between *C.maritimus* and *C.brunneus* can be explained by the difference in the peg number. By contrast, the extremely long file in *C.miramae* is not due to the significant increase in the peg number, but due to the more widely spaced pegs in the distal part of the file.

The long stridulatory files are known in some other species of the *biguttulus* group. *C.biguttuluseuchedickei* from the southern Balkans and north-western Anatolia (Willemse et al. 2009) and *C.maroccanus* Nadig, 1976 from north-western Africa (Ragge and Reynolds 1988), are also characterized by extraordinary long stridulatory files and the widely spaced distal pegs. In *C.brunneusbrevis*Klingstedt 1939 from Southern Finland and north-east Russia (Ragge and Reynolds 1998; Benediktov 2017), the file length is much greater than in the nominate subspecies. In *C.brunneusbrevis*, however, the increased length of the stridulatory file can be explained by the increase in the peg number. In one endemic of the *biguttulus* group in Anatolia, *C.relicticus* Sirin, Helversen & Ciplak, 2010, the peg number was shown to be extremely high (175–225 in male, 194–245 in female; Sirin et al. 2010). Unfortunately, the authors did not measure the length of stridulatory file in *C.relicticus*, but we assume that the file could be also long.

What could be a function of the long stridulatory file? The different parts of the long file can be used during stridulation to produce various song elements (Vedenina et al. 2007; Vedenina and Helversen 2009). This, however, is only evident in *C.biguttuluseuchedickei* (Helversen 1989; Willemse et al. 2009). The calling song of this species consists of 1–3 typical loud echemes, similar to those in the nominate form, that are followed by 1–5 softer aftersongs (quiet parts of the song produced at the end of singing). Aftersongs are produced at a low position of the legs, and presumably the distal pegs are used for sound generation. In *C.miramae*, however, the long stridulatory file does not seem to be specifically involved in sound generation: at least, no song elements were found to be generated by distal pegs only. The leg movements in other species of the *biguttulus* group with a long file or high peg number have not been studied or studied only for certain song types.

It is noteworthy that stridulatory pegs function not only as a mechanic part of the stridulatory apparatus, but also as the mechanoreceptors (Hustert et al. 1999). It was shown in two distantly related species of Gomphocerinae, *C.biguttulus* and *Syrbulamontezuma* (Saussure, 1861), that two sensory cells innervate each peg in themale and each tubercle in the female. These mechanoreceptors can deliver specific proprioceptive information about the contact between the stridulatory file and the vein of the fore wing. A subtle sensory control is required for measuring the pressure of the leg against the wing. The current study of the *C.miramae* songs shows that the loud *maritimus*-like echeme is apparently produced by legs being more pressed to the wings than during the softer *brunneus*-like echeme (Fig. 7). The latter echeme may be also produced with different leg pressure depending on the calling or courtship behavior; during courtship, the sound can be even absent despite the appropriate leg movements (Fig. 8).

In species of the *C.albomarginatus* group, the peg number and density differ only at the proximal parts of the stridulatory files (Vedenina and Helversen 2009). The various species of this group produce different and very conspicuous visual displays in a particular part of the courtship: during the stroke with the hind tibiae, the femora are kept at the extra-high, almost vertical, position. At this moment, the proximal pegs may participate in producing sound. Therefore, the divergence in visual display and the changes in the peg morphology in the *albomarginatus* group could strengthen each other. A similar assumption can be made for the evolution in song and the stridulatory file structure in the *biguttulus* group.

### Peculiarities of the *Chorthippusmiramae* song

The calling song of *C.miramae* is conspicuously different from the songs of *C.brunneus* and *C.maritimus*, by the presence of two types of echemes, which were recorded in 82% of males. In the calling songs of 18% of *C.miramae* males, however, only the *maritimus*-like echemes were recorded. The latter specimens, however, clearly belong to *C.miramae* based on morphology and courtship and rivalry songs.

Until now, the calling songs of *C.miramae* were only presented under the name *C.yersini* by Bukhvalova (1993) and *C.porphyropterus* by Benediktov (2005). Both authors claim the presence of the two echeme types. From the oscillograms presented, one could see many similarities with the songs of *C.maritimus* and *C.brunneus*. The current song analysis that includes not only the sound but also the leg-movement analysis indicates that both *maritimus*-like and *brunneus*-like elements have some peculiarities in the *C.miramae* song. The *maritimus*-like echemes rarely show the distinct pulses within syllable, whereas such pulses in the calling song of *C.maritimus* are typically present. This may be determined by the larger shift between the two legs in *C.miramae* than in *C.maritimus*. The *brunneus*-like echeme in the *C.miramae* song is produced by simple up and down leg-movements, whereas each leg generates a simple upstroke but a two-step downstroke in the *C.brunneus* song. The sound pulses, however, are of a similar temporal structure in both species. The similarities between the calling songs could explain why *C.miramae* is not found together with *C.maritimus* and *C.brunneus* in the same biotopes (despite the latter two species often occur syntopically). According to the concept of ‘acoustic niches’ (Bukhvalova 2006; Tishechkin and Bukhvalova 2009), the combination of the syllable rate and syllable temporal pattern determines the species ‘place’ in the acoustic environment of the grasshopper community. Since these song parameters overlap within the species pairs *C.miramae* / *C.maritimus* and *C.miramae* / *C.brunneus*, the absence of each pair in the same biotope is not surprising.

*C.miramae* generally demonstrates a richer song repertoire than the other two species. The courtship song of *C.miramae* is similar to the *brunneus*-like echeme, but the sound is very soft. In some cases, leg movements of *C.miramae* do not produce any sound at all, which may be interpreted by a female as a visual display. Notably, there is no specific courtship song in both *C.brunneus* and *C.maritimus*. As for a rivalry song, this is present in *C.maritimus* and *C.miramae* but not in *C.brunneus*. The rivalry song of *C.miramae* is similar to that in *C.maritimus*. It comprises the first syllable with distinct pulses lasting longer than the subsequent syllables with fuzzy pulses. More often, however, the rivalry repertoire in *C.miramae* includes short syllables similar to the first one in the *maritimus*-like echeme but repeated at the rate of 2–2.5/s.

In most species of the *biguttulus* group, the rivalry song is similar to the calling song (Ragge and Reynolds 1998). The rivalry song may be shorter than the calling song, but similar in temporal structure and usually does not contain any new elements. Only *C.maroccanus* produces a characteristic rivalry song containing two elements, one element similar to the calling song and the second unique element. Thus, *C.miramae* is another species of this group, in which the rivalry song is principally different from the calling song.

### The relationship of *Chorthippusmiramae* with other members of the *biguttulus* group

It has been suggested that the *biguttulus* group comprises many young, closely related species, some of which may be of hybrid origin. Some species of this group were found to hybridize in nature (e.g., Ragge 1976; Bridle and Butlin 2002; Kleukers et al. 2004; Nolen et al. 2020), whereas some of them were hybridized in laboratory in no-choice conditions and produced viable and fertile offspring (Helversen and Helversen 1975; Gottsberger and Mayer 2007). The similarity of the *C.miramae* song with the songs of *C.brunneus* and *C.maritimus* might suggest a hybrid origin of *C.miramae*.

One of the most well studied hybrid zones within the *biguttulus* group is a hybrid zone between *C.jacobsi* and *C.brunneus* in northern Spain (e.g., Bridle and Butlin 2002; Saldamando et al. 2005; Bridle et al. 2006). The calling song of *C.jacobsi* is similar to the song of *C.maritimus*, but of a shorter duration. Songs of F1, F2 and backcross hybrids between *C.jacobsi* and *C.brunneus* were intermediate between the songs of both parental species in all song parameters (Saldamando et al. 2005). At the same time, no combination of the parental song elements was found in the hybrid songs. Similarly, natural hybrids between *C.maritimus* (named as *C.bornhalmi*) and *C.brunneus* from north-eastern Italy were shown to sing intermediate songs (Kleukers et al. 2004). In European Russia and Ukraine, these two species often occur in the same biotope allowing them to hybridize. We suggest that the *C.brunneus* song with unusually long echeme duration and low echeme and pulse rate recorded from loc. 40 (Table 2) may be also attributed to the hybrid. F1 hybrids between *C.maritimus* and *C.brunneus* bred in our laboratory only revealed intermediate songs (unpublished data). It is therefore unlikely that *C.miramae* could evolve from the hybrids between *C.maritimus* and *C.brunneus*.

The number of stridulatory pegs in hybrids between *C.jacobsi* and *C.brunneus* (Saldamando et al. 2005) and *C.brunneus* and *C.bornhalmi* (Kleukers et al. 2004) were shown to be also intermediate between those in parental species. In *C.miramae*, the peg number is similar to that of *C.maritimus*, but significantly larger than that in *C.brunneus*.

Other results were obtained for hybrids between *C.biguttulus* and *C.mollis* (Helversen and Helversen 1975) and *C.brunneus* and *C.biguttulus* (Gottsberger and Mayer 2007). A combination of the parental song elements and even novel song elements were found in hybrids between *C.biguttulus* and *C.mollis*. Thus, the hybrid song may be considered as more complex in comparison with the parental songs. In hybrids between *C.albomarginatus* and *C.oschei* (unrelated species to the *biguttulus* group), the values of several song parameters were significantly larger or smaller than those in the parental songs (Vedenina et al. 2007). Notably, the leg-movement patterns appeared to be simpler in hybrids than these in both parentals. In hybrids between *C.brunneus* and *C.biguttulus*, the species-specific syllable structure was largely lost, because the leg-movement patterns were also simplified in comparison to the parental patterns (Gottsberger and Mayer 2007). These divergences in inheritance of different song parameters are likely the result from incompatibility of neuronal networks that control stridulatory leg movements in hybrids. This hypothesis was offered by Helversen and Helversen (1975). They suggested the two pattern-generating neuronal networks to be formed in the central nervous system of hybrids because of nonhomology of the parental elements. The outputs of the two networks converge in a common final pathway, probably at the level of the motoneurons, and may lead to the superimposed pattern of the hybrid song. In *C.brunneus*, *C.biguttulus*, and *C.mollis*, the song elements in terms syllable structure are suggested to be nonhomological. In *C.biguttulus*, for example, the first and the loudest pulse in each syllable is generated by an accentuated downstroke of the legs; each syllable is usually produced by three up-and-down leg movements; the two legs moving in slightly different patterns (e.g., Elsner 1974; Helversen and Helversen 1983, 1994). It is no coincidence that some authors attribute these three species to different subgroups of the *biguttulus* group (Willemse et al. 2009; Sirin et al. 2010). By contrast, syllable of the calling song in *C.brunneus*, *C.jacobsi* and *C.maritimus* (= *brunneus* subgroup) is produced by similar leg movements (simple upstroke and stepwise downstroke) and may be considered as homological element. It is therefore not surprising why hybrids between the species within the *brunneus* subgroup generate purely intermediate songs without novel elements or combination of the parental elements.

Considering all the aforesaid, what can we say about the origin of *C.miramae*? We hypothesize that this species could have evolved as a result of hybridization between other species of the *biguttulus* group, for example, between *C.biguttulus* and *C.maritimus*. The two species are vicariant: the first one occurs in the north, the second one – in the south. For example, in the Ukraine *C.biguttulus* is found more in the north, whereas *C.maritimus* more in the south. Eastwards, this border is shifting, the ranges overlap, and the species may occur syntopically. In the latter case, however, *C.maritimus* can be found in the first half of summer, whereas *C.biguttulus* – in the second half of summer. This indicates that the species tend not to meet, probably because the syllable rate in calling songs is quite similar; the syllable structure, however, is very different. Meanwhile, we do not exclude that hybridization may occur between these species when one of them is rare and another is abundant. To date, no laboratory hybrids were bred between them, and nothing is known about *biguttulus*×maritimus hybrid song. The hybridization experiments between these species could be a subject of future studies.

We also hypothesize that *C.miramae* could diverge from *C.maritimus*. The latter species is widespread in Anatolia, where it occurs in highlands, thus forming isolated populations. In Anatolia, there is also another species of the *biguttulus* group, *C.relicticus*, occurring very locally in the Southern Anatolian Taurus (Sirin et al. 2010). Its calling song is similar to the *brunneus*-like echeme of *C.miramae*, which is produced by simple up and down strokes of the legs moving in antiphase. Sirin et al. (2010) suggest that this species could have radiated from a *C.maritimus* (named as *C.bornhalmi* in the paper) like ancestor in an interglacial refugium. In southern territories, the members of the *biguttulus* group, being the cold-resistant species, are suggested to be isolated during interglacial periods and spread down and expanded their ranges during glacial periods. If we suggest the divergence of *C.miramae* from *C.maritimus*, the spreading of the former to the north could occur, on the contrary, during interglacial periods.

To test both hypotheses (hybrid origin of *C.miramae* or its divergence from a *C.maritimus*-like ancestor in a glacial refugium), it is necessary to conduct genomic studies. A recent analysis of mitochondrial and nuclear genomes in the *biguttulus* group in Western Europe (Nolen et al. 2020) shows that four species, *C.brunneus*, *C.biguttulus*, *C.rubratibialis* and *C.mollis*, experienced a long period of geographic isolation, followed by secondary contact and extensive introgression. According to Nolen et al. (2020), *C.mollis* was the first species to split, *C.biguttulus* was the next, followed by *C.rubratibialis* and *C.brunneus*. Mitochondrial genomes suggest that the radiation is relatively recent, dating to the mid-Pleistocene. Thus, the species of the *biguttulus* group must have experienced multiple episodes of contraction and expansion during the multiple glacial periods that affected the European continent. Taking this into account, it would be especially interesting to sample other species of the *biguttulus* group, especially those at or near the described refugia in Eurasia.

## Supplementary Material

XML Treatment for Chorthippus brunneus

XML Treatment for Chorthippus maritimus

XML Treatment for Chorthippus miramae
